# Bone marrow mesenchymal stem cells promote osteosarcoma cell proliferation and invasion

**DOI:** 10.1186/s12957-015-0465-1

**Published:** 2015-02-15

**Authors:** Fu-Xiang Yu, Wei-Jian Hu, Bin He, Yi-Hu Zheng, Qi-Yu Zhang, Lin Chen

**Affiliations:** Department of General Surgery, The First Affiliated Hospital of Wenzhou Medical University, Nan Bai Xiang Street, Ouhai District, Wenzhou, Zhejiang 325002 China; Department of Pathology, The First Affiliated Hospital of Wenzhou Medical University, Nan Bai Xiang Street, Ouhai District, Wenzhou, Zhejiang 325002 China

**Keywords:** Bone marrow mesenchymal stem cells, Osteosarcoma

## Abstract

**Background:**

Bone marrow-derived stem cells (BMSCs) are locally adjacent to the tumor tissues and may interact with tumor cells directly. The purpose of this study was to explore the effects of BMSCs on the proliferation and invasion of osteosarcoma cells *in vitro* and the possible mechanism involved.

**Methods:**

BMSCs were co-cultured with osteosarcoma cells, and CCK-8 assay was used to measure cell proliferation. The ELISA method was used to determine the concentration of stromal cell-derived factor-1 (SDF-1) in the supernatants. Reverse transcription polymerase chain reaction (RT-PCR) was performed to detect the expression of CXCR4 in osteosarcoma cells and BMSCs. Matrigel invasion assay was performed to measure tumor cell invasion.

**Results:**

SDF-1 was detected in the supernatants of BMSCs, but not in osteosarcoma cells. Higher CXCR4 mRNA levels were detected in the osteosarcoma cell lines compared to BMSCs. In addition, conditioned medium from BMSCs can promote the proliferation and invasion of osteosarcoma cells, and AMD3100, an antagonist for CXCR4, can significantly downregulate these growth-promoting effects.

**Conclusions:**

BMSCs can promote the proliferation and invasion of osteosarcoma cells, which may involve the SDF-1/CXCR4 axis.

## Background

It has been increasingly recognized that cancer cells actively recruit stromal cells into tumors and this recruitment is essential for the generation of a microenvironment that promotes tumor growth [1]. Since the stroma cells can stimulate tumor growth and invasiveness, stromal cells play an active role in cancer progression, induce chemotherapy resistance, and inhibit cancer cell apoptosis [2-5]. Organs have abundant stroma surrounding them, and tumor-stroma interactions play a critical role in tumor progression [6]. Although the exact mechanisms of tumor-stroma interactions remain unclear, some chemokines, including hepatocyte growth factor, epidermal growth factor, vascular endothelial growth factor, and stromal cell-derived factor-1 (SDF-1), have been shown to be involved in the interaction [7,8].

SDF-1 belongs to the CXC chemokine family and is a ligand for CXCR4. SDF-1 is expressed in stromal cells [9], and CXCR4 expression has been found in many cancer types, including breast, ovary, kidney, prostate, and stomach cancer tissue and cell lines [10-12]. In addition, it has been demonstrated that the SDF-1/CXCR4 receptor ligand system plays an important role in carcinoma progression by promoting tumor cell migration and angiogenesis [13,14].

The interactions between mesenchymal stem cells from bone marrow and malignant tumors from the breast, prostate, ovaries, and stomach have been recently described [10,15]. Bone marrow-derived stem cells (BMSCs) are locally adjacent to the tumor tissues and may interact with tumor cells directly. However, the ability of BMSCs to promote tumor cell proliferation remains controversial. It has been reported that BMSCs can promote breast, prostate, non-small lung, and glioblastoma cancer cell growth *in vivo* [16-18]. In contrast, several reports have shown an anti-tumor effect of mesenchymal stem cells. Khakoo *et al.* used systemic injection of mesenchymal stem cells to inhibit the growth of a subcutaneous Kaposi sarcoma xenotransplant [19]. Moreover, the co-implantation of breast cancer cells with mesenchymal stem cells results in tumor growth inhibition and a reduction of metastasis *in vivo* [20]. However, the impact of BMSCs, which are a type of local mesenchymal stem cell, on proliferation and invasion of osteosarcoma has not been reported to date. Therefore, in this study, we determined whether BMSCs can promote the growth and invasion of osteosarcoma and sought to explore the mechanism responsible for these observed effects.

## Methods

### Cell lines and reagents

Human osteosarcoma cell lines MG-63 and OS732 were purchased from the Chinese Academy of Sciences (Shanghai, China). Dulbecco’s modified Eagle’s medium (DMEM) and fetal bovine serum (FBS) were provided by Gibco (Grand Island, NY, USA), and recombinant human CXCL12 (SDF-1) was purchased from R&D systems (Minneapolis, MN, USA). AMD3100, a chemokine receptor antagonist for CXCR4, and Matrigel were obtained from Sigma-Aldrich (St. Louis, MO, USA). The fluorochrome-conjugated antibodies used for immunostaining - anti-CD45-APC, anti-CD29-PE, and anti-CD90-FITC - and appropriate negative controls were from BD (San Diego, CA, USA).

### Isolation of human BMSCs

Bone marrow was obtained from healthy persons who had provided written informed consent. This process was approved by the institutional review board of the First Affiliated Hospital of Wenzhou Medical University. A solution of density of 1.073 g/mL by dilution of Percoll was added to the bottom of the separating tube. Then, the fresh bone marrow of 20 mL was added to Percoll in a volume ratio of 1:1 gently. Centrifugation was carried out at room temperature at 3,000 rpm for 30 min. The white cell band between the two layers was transferred, and the pelleted cells were washed two times with the medium without FBS. Finally, cells were resuspended and grown in low-glucose (1,000 mg/L) DMEM (L-DMEM) containing 20% FBS, 100 μg/L penicillin, and 100 μg/L streptomycin in a humidified environment with 5% CO_2_ at 37°C. After 48 h, unattached cells were washed and removed. The cells were then grown in a humidified incubator at 37°C for an additional 4 weeks. Before phenotype analysis by flow cytometry, cells were fixed and permeabilized by a Cytofix/Cytoperm reagent (Becton Dickinson PharMingen, San Jose, CA, USA) after being harvested from six-well assay plates. Then, they were indicated by a panel of antibodies including PE-conjugated CD29 antibody, FITC-conjugated CD90 antibody, and APC-conjugated CD45 antibody.

### Differentiation of human BMSCs into adipocytes

BMSCs were cultured to confluence in 35-mm dishes containing DMEM. The medium was then removed and fresh DMEM was added containing 0.5 mM IBMX, 1.0 μM dexamethasone, and 300 nM insulin. The cells were cultured in the differentiation medium for 2 days, and then the medium was changed every 2 days with DMEM containing only 300 nM insulin for a total of two times. After this step, the cells were incubated in DMEM without any additives, which was changed every 10 days. Fully differentiated adipocytes were observed by light microscopy based on morphology. Oil red O staining was used to detect fat droplets for the various treatments as described above.

### Transwell co-culture system and CXCR4 antagonist treatment

BMSCs were cultured in apical compartments of transwells (transwell insert 0.4 μm; Millipore, Billerica, MD, USA) with osteosarcoma cells grown in the basal compartment of the plate (Millipore). BMSCs were seeded onto the upper layer of transwells without direct contact with osteosarcoma cells. Osteosarcoma cells were seeded onto the lower layer of transwells. CXCR4 antagonist, AMD3100 (100 ng/mL), was added into the wells when drug experiment was needed. All cells were grown in L-DMEM supplemented with 10% FBS at 37°C in 5% CO_2_. As a control, the same osteosarcoma cells were seeded instead of BMSCs onto the upper layer. After culturing the cells for 72 h, cell proliferation was analyzed using a CCK-8 kit (Dojindo, Kumamoto, Japan) following the manufacturer’s instructions.

### Proliferation assay

The CCK-8 kit solution was added to each well, the cells were incubated for another 2 h, and the absorbance at 450 nm was measured by using a spectrophotometer. The amount of the formazan dye, generated by the activities of dehydrogenases in cells, is directly proportional to the number of living cells. The proliferation rate (%) of cells was calculated using the following equation: 1 − (*A* of control − *A* of BMSCs) / (*A* of control − *A* of osteosarcoma cells) × 100%.

### Invasion assay

Invasion assay was performed using Boyden chambers with inserts (pore size: 8 μm) coated with Matrigel in 24-well plates. Briefly, osteosarcoma cells (4 × 10^5^ cells/mL) were suspended in 250 μL medium containing 2% FBS in the upper chamber, which was pre-coated with 15 μL Matrigel (0.5 μg/mL). BMSCs were seeded onto the lower layer of transwells at a density of 2 × 10^5^ cells/well. The co-culture system was incubated at room temperature for 15 min. The control group consisted of plates with medium containing 2% FBS and osteosarcoma cells in the upper chamber only. The plates were incubated for 24 h at 37°C. Cells on the upper side of the filters were removed with cotton-tipped swabs, and invaded cells were fixed with 4% paraformaldehyde, washed with PBS, air dried, and stained with crystal violet for 30 min. They were then rinsed several times with distilled water. The number of invading cells was counted in five random microscopic fields.

### ELISA assay

After culturing cells for 72 h at 37°C containing 5% CO_2_ in a humidified incubator, the culture medium was collected separately and centrifuged at 1,000 rpm for 5 min to remove debris. The supernatants were then frozen at −80°C for further assessment by ELISA. ELISA was performed using the human CXCL12/SDF-1 kit (R&D Systems, Minneapolis, MN, USA) according to the manufacturer’s instructions.

### Reverse transcription polymerase chain reaction

Total RNA was extracted from cultured cells using Trizol (Invitrogen, Carlsbad, CA, USA). The cDNA was synthesized using a random primer from 1 μg of total RNA with the Revert Aid First Strand cDNA Synthesis Kit according the to manufacturer’s instructions (Fermentas, Glen Burnie, MD, USA). The following primers were used for reverse transcription polymerase chain reaction (RT-PCR): human SDF-1 (sense, 5′-gctttgagtgactgggtt-3′; antisense, 5′-gtggcaagatgatggttt-3′), PCR product size: 124 bp; human CXCR4 (sense, 5′-gaagctgttggctgaaaagg-3′; antisense, 5′-gagtcgatgctgatcccaat-3′), PCR product size: 345 bp; and human beta-actin (sense, 5′-actcttccagccttccttc-3′; antisense, 5′-tgtcaccttcaccgttcc-3′), PCR product size: 516 bp. PCR was performed following the manufacturer’s instructions. The cycling conditions were 3 min at 94°C, followed by 30 cycles of denaturation at 94°C for 30 s, annealing at 54°C for 30 s, and extension at 72°C for 60 s. Amplified DNA fragments were resolved by electrophoresis on 1% agarose gels containing ethidium bromide.

### Statistical analysis

Data were expressed as means ± standard deviation (SD) and analyzed using analysis of variance (ANOVA). *P* < 0.05 was considered statistically significant.

## Results

### Identification of BMSCs

**Figure 1:**
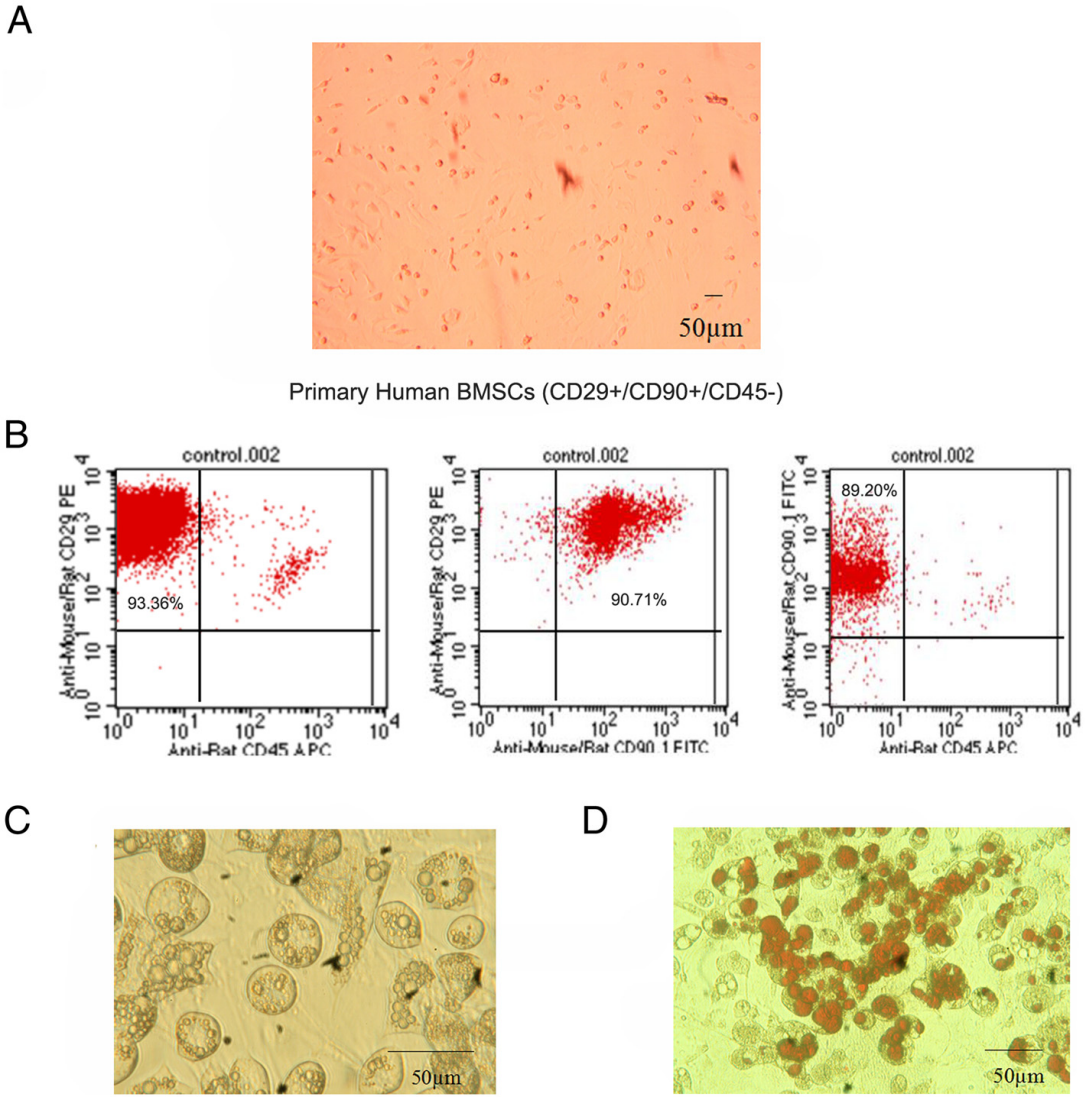
**Identification of bone marrow-derived stem cells. (A)** The phenotype of BMSCs isolated from human bone marrow tissue (on the first day; 100× magnification fold). **(B)** The expression of CD45, CD29, and CD90 in isolated BMSCs. **(C)** The fat droplets were observed in the cytoplasm of adipocytes at 400× magnification. **(D)** The fat droplets were stained with Oil red O. Image is magnified 200 ×.

Primary human BMSCs were spindle-shaped and adherent to the dish (Figure 1A). Several BMSC markers were confirmed by flow cytometric analysis, including CD29, CD90, and CD45 (97.22 ± 1.94, 94.91 ± 3.88, and 2.22 ± 0.21% positivity, respectively) (Figure 1B). The CD marker analysis of BMSCs was consistent with typical mesenchymal stem cells. After differentiation, the accumulated adipocytes in BMSCs were detected by oil red O staining for the various treatments. Most of the BMSCs differentiated into adipocytes successfully, and Oil red O staining detected numerous fat droplets in the cytoplasm of adipocytes.

### Effect of BMSCs on the proliferation and invasion of osteosarcoma cells

**Figure 2:**
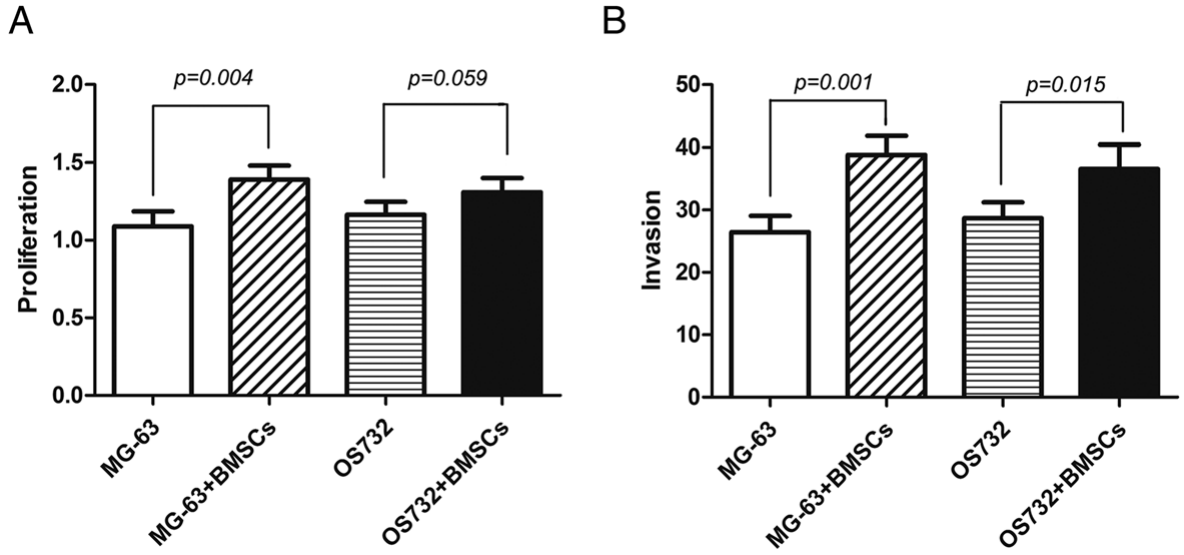
**Effect of BMSCs on the proliferation and invasion of osteosarcoma cells. (A)** After co-culturing BMSCs with osteosarcoma cells for 72 h, the proliferation of osteosarcoma cells was examined using the CCK-8 assay. The proliferation of MG-63 and OS732 cells co-cultured with BMSCs was significantly increased compared to that of the respective cells cultured alone. **(B)** BMSCs were co-cultured with osteosarcoma cells for 72 h, and the percentage of osteosarcoma cell invasion was examined under a microscope. The invasion of MG-63 and OS732 cells was significantly greater in the presence of BMSCs compared to control cells.

Using a cell co-culture system, we found that BMSCs promote the proliferation of osteosarcoma cells (Figure 2A). The number of osteosarcoma MG-63 and OS732 cells cultured with BMSCs in the system was significantly higher than the cells grown in the absence of BMSCs. Moreover, the invasion of osteosarcoma cells incubated with the BMSC supernatant was enhanced (Figure 2B).

### Secretion of SDF-1 by BMSCs

**Figure 3:**
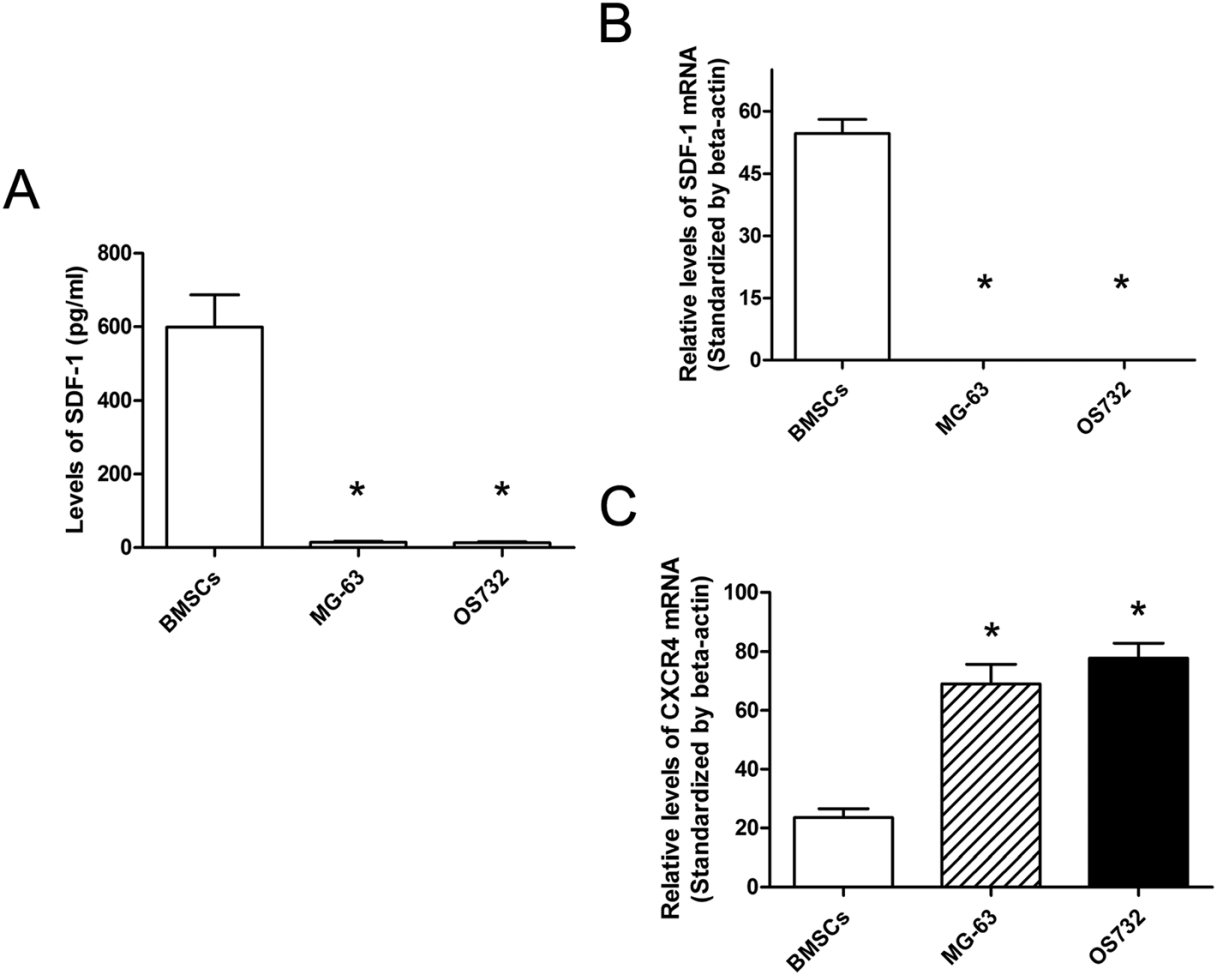
**SDF-1 secretion and SDF-1 and CXCR4 expression. (A)** The SDF-1 concentration in the BMSC supernatant as detected by ELISA. BMSCs had significantly higher levels of SDF-1 compared to MG-63 and OS732 cells. **(B)** The RT-PCR analysis of SDF-1 mRNA expression in osteosarcoma cell lines and BMSCs. **(C)** The RT-PCR analysis of CXCR4 mRNA expression in osteosarcoma cell lines and BMSCs. (*P < 0.01 compared to the BMSCs).

The amounts of SDF-1 secreted by BMSCs and osteosarcoma cells were determined by ELISA. The concentration of SDF-1 in the BMSC supernatant was nearly 600 pg/mL. Importantly, a very low concentration of SDF-1 was detected in the supernatant of osteosarcoma cells (Figure 3A). These data confirmed that BMSCs, but not osteosarcoma cell lines, secret SDF-1.

### Expression of SDF-1 and CXCR4 in cultured osteosarcoma cell lines and BMSCs

We evaluated the expression of SDF-1 and CXCR4 by RT-PCR in cultured cells. BMSCs exhibited a higher expression of SDF-1 compared to MG-63 and OS732 cells (Figure 3B). However, these two osteosarcoma cell lines showed a higher expression of CXCR4 compared to BMSCs (Figure 3C).

### Effects of SDF-1 on the proliferation of osteosarcoma cell lines

**Figure 4:**
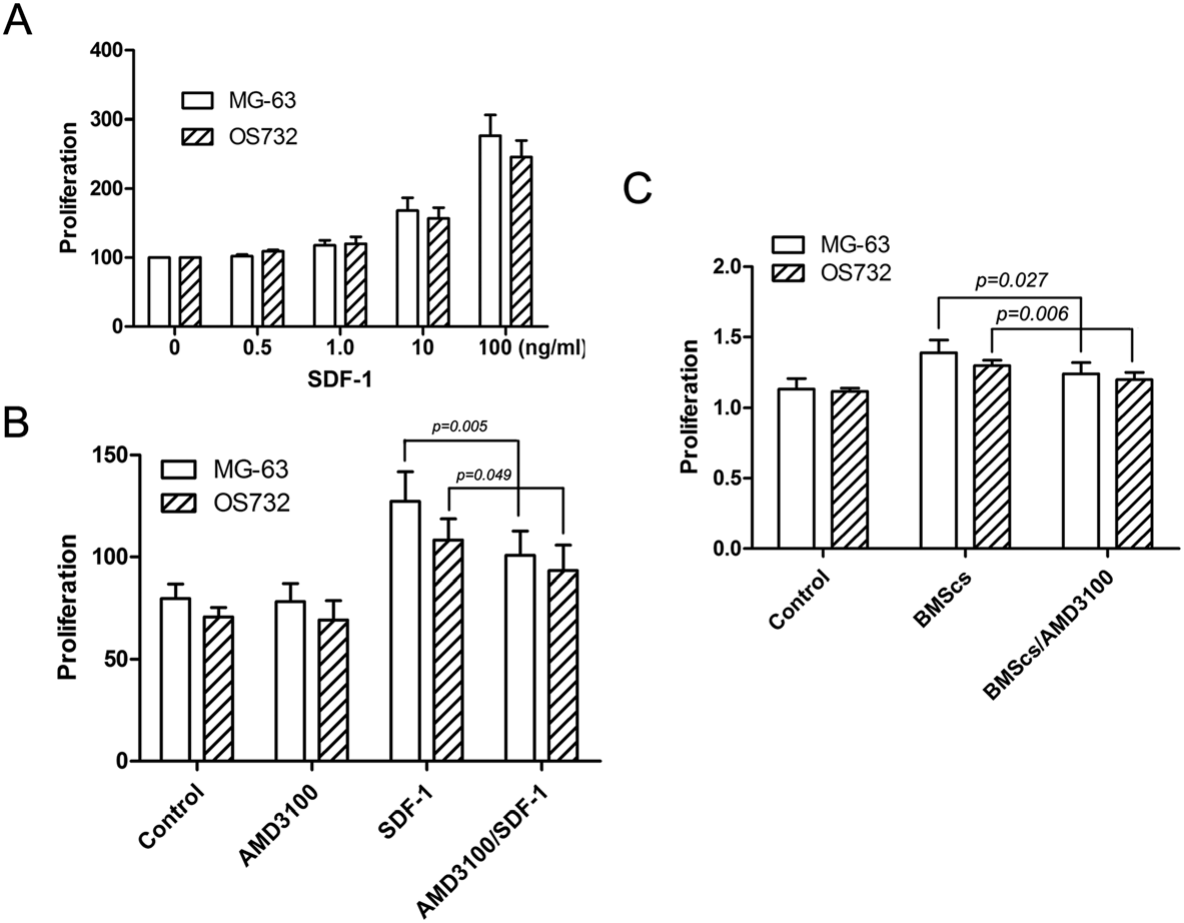
**Effects of SDF-1 on the proliferation of osteosarcoma cell lines. (A)** SDF-1 promotes the proliferation of osteosarcoma cells in a dose-dependent manner. **(B)** AMD3100 partially blocks SDF-1-mediated proliferation. **(C)** AMD3100 downregulates the effects of BMSCs on osteosarcoma cell line proliferation.

SDF-1 enhanced the proliferation of osteosarcoma cells in a dose-dependent manner compared to controls (Figure 4A). The CXCR4 antagonist AMD3100 significantly blocked SDF-1-induced proliferation (*P* < 0.05) but did not inhibit MG-63 and OS732 basal cell growth in the absence of SDF-1 (Figure 4B).

### Effects of BMSCs on the proliferation of osteosarcoma cells in the presence of AMD3100

The effect of BMSCs on MG-63 and OS732 cell growth in the presence of AMD3100 was also determined using the CCK-8 assay. We found that BMSCs enhanced the proliferation of both osteosarcoma cell lines, which were significantly reduced by the addition of AMD3100 in a dose-dependent manner (*P* < 0.05) (Figure 4C).

## Discussion

The interaction between the tumor and tumor-associated stroma has been reported in many studies, and the importance of the local tumor microenvironment for tumor progression has been recognized for many years [21,22]. This study demonstrated that BMSCs promote osteosarcoma cell proliferation and invasion *in vitro*. Furthermore, we demonstrated that the SDF-1/CXCR4 axis plays an important role in mediating the tumor-promoting effect.

Since BMSCs are derived from bone marrow tissue that is abundant in the tissues adjacent to osteosarcoma, it is very important for their capacity to promote osteosarcoma cell growth. We first found that BMSCs, but not osteosarcoma cells, secret SDF-1. SDF-1 is a secreted chemokine that is released into the interstitial space, where it acts on cells in the local microenvironment in a paracrine fashion to stimulate directional migration of hematopoietic and nonhematopoietic normal and malignant cells [23]. SDF-1 exerts a variety of biological functions, including the regulation of angiogenesis and inhibition of apoptosis as well as tumor growth, migration, and invasion promoting effects through the SDF-1/CXCR4 receptor ligand axis [24-26]. Furthermore, Brand *et al.* demonstrated that SDF-1 stimulation induces a significant increase in VEGF protein levels in the colorectal cancer line HT-29 [27]. SDF-1 has also been demonstrated to play a role in tumors, including breast cancer, melanoma, ovarian cancer, gastric cancer, and other carcinomas [12].

We also assessed the expression of CXCR4 in the two osteosarcoma cell lines using RT-PCR and found that the mRNA levels were elevated compared to controls. In contrast, CXCR4 mRNA levels were very low in BMSCs. Importantly, these results were consistent with previous studies [13,28]. We also found that the SDF-1/CXCR4 axis plays an important role in the proliferation of osteosarcoma cells. In this study, we demonstrated that recombinant SDF-1 could significantly promote the proliferation of osteosarcoma cells. Blocking the receptor with AMD3100 was sufficient for preventing SDF-1-mediated proliferation in MG-63 and OS732 cells. It has been further reported that CXCR4 signaling in osteosarcoma cell lines can promote MG-63 and OS732 proliferation [29]. Taken together, therefore, these data suggest that CXCR4 is a potential therapeutic target in osteosarcoma.

Moreover, we found that BMSCs stimulate the invasive behavior of osteosarcoma cells. An invasion assay using a Matrigel-coated invasion chamber showed that BMSCs stimulated invasion of osteosarcoma cells and that these effects were inhibited by the addition of the CXCR4 inhibitor AMD3100. Therefore, SDF-1 plays a critical role in promoting the invasion of osteosarcoma cells. However, although the BMSC-induced proliferation was inhibited by AMD3100, proliferation was still higher than the control. Therefore, other factors, such as matrix metalloproteinase-9 (MMP-9), MMP-2, and vascular endothelial growth factor [10,30], may also be involved in promoting the proliferation and invasion of osteosarcoma cells. It has been shown that myofibroblast-derived SDF-1 recruits endothelial progenitor cells to sites of carcinomas, thereby enhancing angiogenesis and tumor growth [31]. Therefore, BMSC-derived SDF-1 may be partially responsible for the proliferation and invasion of osteosarcoma cells.

## Conclusions

The aim of this study was to gain a better understand of the mechanisms related to osteosarcoma growth and invasion. Despite advances in surgical and medical therapies, overall survival rate for patients with osteosarcoma remains low [32,33]. Therefore, a better understanding of the fundamental nature of this cancer is needed to improve clinical outcome. Our finding that local stem cells adjacent to osteosarcoma cells are involved in tumor growth and invasion may be useful for osteosarcoma therapy, as these results suggest that the bone marrow tissue surrounding the osteosarcoma tissue should be resected completely. Future studies need to confirm these findings.
